# Effect of Parasitic Infections on Hematological Profile, Reproductive and Productive Performance in Equines

**DOI:** 10.3390/ani15223294

**Published:** 2025-11-14

**Authors:** Abd Ullah, Mingyang Geng, Wenting Chen, Qifei Zhu, Limeng Shi, Xuemin Zhang, Muhammad Faheem Akhtar, Changfa Wang, Muhammad Zahoor Khan

**Affiliations:** 1 College of Agriculture and Biology, Liaocheng University, Liaocheng 252000, China; 2 Yili Kazak Autonomous Prefecture Livestock General Station, Xinjiang Autonomous Region, Yili 835000, China

**Keywords:** *Trypanosoma evansi*, *Theileria equi*, *Babesia caballi*, *Cyathostomins*, vector-borne parasites, equines fertility, hematobiochemical profile, milk composition, meat safety, zoonotic transmission

## Abstract

Equines play a crucial role in global food security, economic development, and recreation, particularly in regions such as Central Asia, parts of Africa, and South America. However, parasitic infections significantly impact their health, productivity, and reproductive performance, leading to economic losses and reduced animal welfare. This review synthesizes the effects of parasitic infections, including protozoan, helminthic, and ectoparasitic species, on equines. These infections cause hematological alterations like anemia, leukocytosis, leukopenia, and thrombocytopenia, compromising overall health and resilience. Protozoan parasites, such as *Trypanosoma* spp., *Theileria equi*, and *Babesia caballi*, directly affect semen quality and fertility by causing testicular lesions, orchitis, and hormonal disruptions. Helminths like *Cyathostomins* and *Strongyles* reduce nutrient absorption, impairing productivity, while some protozoan species can cause abortion through transplacental transmission. Zoonotic parasites, including *Sarcocystis* spp. and *Toxoplasma gondii*, pose a human health risk through contaminated meat and milk consumption. Despite the effectiveness of conventional anthelmintics, emerging biological control methods like Duddingtonia flagrans (BioWorma^®^ and Bioverm^®^) show promise. However, the development of standardized herbal anthelmintics and vaccines is hindered by limited efficacy validation, complex parasite biology, and inadequate funding. The need for better diagnostic tools and sustainable treatments remains critical for the long-term sustainability of the equine industry.

## Simple Summary

This comprehensive review examines how parasitic infections compromise equine health across multiple biological systems. The manuscript systematically evaluates parasitological diversity in equines, encompassing protozoans, helminths, and ectoparasites, and their multifaceted consequences. Key areas addressed include hematological disturbances, impairment of male and female reproductive function, pregnancy complications including abortion risks, and deterioration of animal-derived food products. The review integrates current knowledge on transmission pathways, diagnostic methodologies, and therapeutic interventions while highlighting zoonotic risks associated with equine parasites, particularly through meat and milk consumption. By synthesizing 217 peer-reviewed publications spanning 2000–2025, this work identifies critical knowledge gaps and emphasizes the necessity for integrated parasite management strategies to safeguard equine welfare, enhance production efficiency, and protect public health.

## 1. Introduction

Equines play a multifaceted and indispensable role in human livelihoods across the globe, contributing significantly to food security, economic development, and recreational activities [1,2,3]. In many regions, particularly in Central Asia, parts of Africa, and South America, equine milk serves as a valuable source of nutrition, being rich in proteins, vitamins, and minerals, while also possessing medicinal properties that are highly valued in traditional medicine [4,5,6,7,8,9]. Similarly, equine meat production represents an important protein source in various cultures and contributes to household income in pastoral and agricultural communities [10,11,12,13,14]. Beyond food production, equine skin and hides are essential raw materials for the leather industry, providing durable materials for clothing, footwear, and various handicrafts [15,16,17]. While equines make substantial contributions to human livelihoods, their health and productivity are significantly compromised by disease burdens, particularly parasitic and viral infections, which pose major constraints to their optimal performance and welfare.

Parasitic infections are widespread in equines, posing a significant threat to their health and productivity, with far-reaching consequences on reproductive efficiency and product quality [18,19]. The infections frequently induce significant hematological disruptions, including polycythemia, anemia (mild, moderate, or severe), leukocytosis (mild, moderate, or severe), leukopenia, and platelet abnormalities like thrombocytopenia and, rarely, thrombocytosis. These changes, along with physiological, stress, or inflammatory leukogram variations, collectively impair health and performance [20]. Beyond these systemic effects, emerging evidence suggests that parasitic burdens critically influence reproductive efficiency, semen quality, and fertility in equines [21,22], while also altering the quality and yield of milk [23]. The presence of these parasites reduces the nutritional value of the meat, making it less desirable for consumption [24].

The hematological changes associated with parasitism, such as those caused by Equine parasitic infections like *Trypanosoma evansi*, *Theileria equi*, and *Babesia caballi*, often result in anemia, reflected by reduced red blood cells (RBCs), hematocrit (HCT), and hemoglobin (Hb), alongside increased mean corpuscular volume (MCV), mean corpuscular hemoglobin (MCH), and mean corpuscular hemoglobin concentration (MCHC), indicating altered red blood cell morphology [25,26,27,28]. Leukocyte changes vary by parasite and infection stage, with common thrombocytopenia in piroplasma infections and shifts in monocytes, eosinophils, and lymphocytes [26,27]. Such hematological disturbances compromise oxygen delivery, exacerbate metabolic stress, and impair physiological resilience, ultimately reducing work capacity, reproductive performance, and production efficiency in affected animals.

In male equines, parasitic infections may directly or indirectly impair testicular function and disrupt spermatogenesis, leading to compromised semen parameters such as reduced motility, viability, and concentration [29,30]. While in females, parasites such as *Trypanosoma evansi* cause chronic parasitic infections, which can disrupt Vaginal/Vulvar Hemorrhages, estrous cycles, and conception rates [30,31]. While this study in cattle demonstrates that nematode infections (e.g., Ostertagia) reduce milk production by disrupting growth hormone (GH) and IGF-I levels [32], in equine Sarcocystosis, the muscle tissue is affected, thereby reducing the nutritional value of the meat [24]. Thus, parasitic infections in equines not only disrupt hematological parameters but also impair reproductive efficiency in males by compromising semen quality and in females by altering estrous cycles, conception rates, and overall fertility, ultimately diminishing breeding success.

This review outlines the specific impacts of parasitic infections on equine semen quality, milk composition, meat yield and safety, as well as their crucial implications for pregnancy outcomes in equids. Understanding these multifaceted effects is vital for formulating effective parasite control strategies that not only reduce economic losses and improve animal welfare but also identify research gaps and guide improved management approaches to enhance equine health and productivity.

## 2. Methodology for Literature Search

This review systematically investigates the impact of parasitic infections on hematological alterations and their consequences for reproductive performance, pregnancy outcomes, and milk and meat production in equines. To ensure comprehensive coverage and scientific rigor, a structured literature search was conducted across four major databases—PubMed, Web of Science, Scopus, and Google Scholar. The search targeted peer-reviewed articles published between 2000 and 2025, thereby encompassing both classical foundational studies and the most recent scientific advances. Relevant studies were identified using specific keywords, including equine parasites, hemoparasites, gastrointestinal nematodes, hematological changes, semen quality, stallion fertility, equine pregnancy, abortion, milk production, meat quality, and zoonotic transmission. Reference lists of key papers were also screened manually to identify additional relevant studies not captured in the initial search.

A total of 350 records were initially retrieved. After careful screening of titles, abstracts, and full texts against the eligibility criteria, 257 articles were finally included in this review. Articles were selected based on predefined inclusion and exclusion criteria to ensure methodological consistency and scientific rigor. Inclusion criteria encompassed original research articles and review papers focused on equine parasitic infections, hematological alterations, reproductive efficiency, productivity traits, and zoonotic implications. Studies addressing diagnostic, therapeutic, or control strategies were also included. Exclusion criteria comprised conference abstracts, book chapters, unpublished data, non-indexed or non-peer-reviewed sources, and non-English publications.

## 3. Parasitological Classification of Equine Parasites and Their General Effects

Parasitic infections in equines represent a significant constraint on hematological profile, health, productivity, and reproductive performance [19,26,33,34]. These parasites can be systematically classified into three major groups: protozoa, helminths, and ectoparasites [35,36], each exerting distinct impacts on hematological health, reproductive efficiency, and productivity [19,26,33,34]. Each group comprises species with distinct pathogenic mechanisms that disrupt hematological balance, weaken the immune system [37,38], and interfere with reproductive physiology, ultimately reducing productivity [39,40], work capacity [40], and overall productivity [24]. Understanding their taxonomic classification and zoonotic potential is crucial for developing effective control strategies and safeguarding both animal and public health.

### 3.1. Protozoan

Protozoan parasites are single-celled organisms that can infect equines in various organs, including the blood, gastrointestinal system, and muscles [35,41,42,43]. Some of the most significant protozoan parasites in equines include *Trypanosoma* spp., *Theileria* spp., *Babesia* spp., and *Sarcocystis* spp. [19,44,45,46]. *Trypanosoma evansi*, the causative agent of surra, induces severe anemia, fatigue, weight loss, and immune suppression in equines, significantly impairing their health, reproductive performance [47]. *Theileria* spp. and *Babesia* spp., both protozoan parasites transmitted by ticks, cause piroplasmosis in equines, leading to anemia, fever, jaundice, thrombocytopenia, and impaired fertility, which collectively reduce the animal’s overall health and performance [48,49]. Coccidiosis, caused by the protozoan *Eimeria* spp., is an occasional gastrointestinal infection in horses. It can lead to diarrhea, poor growth, and malnutrition, particularly in young horses, impairing nutrient absorption. This infection significantly affects equine health, reducing productivity and leading to economic losses in equine husbandry [50,51,52,53]. These protozoa can cause a variety of health issues, from mild illness to severe systemic infections. They are known to cause anemia, immune suppression, and oxidative stress, which significantly impact the overall health and performance of affected animals.

### 3.2. Helminths (Nematodes, Trematodes, Cestodes)

Helminths, or parasitic worms, are among the most common parasites in equines. These worms include nematodes (roundworms), trematodes (flukes), and cestodes (tapeworms) [35,54,55,56]. Among trematodes, *Fasciola hepatica* infection has been reported in equines, causing hepatic damage, anemia, and reduced productivity [57,58]. Filarial worms such as *Setaria equina* may inhabit the peritoneal cavity, scrotum, or spermatic cord, occasionally inducing orchitis, edema, and granulomatous inflammation that can compromise reproductive health [29,59]. Another study also reported that in South Korea, *Setaria digitata* has been identified as the principal etiological agent of equine neurological ataxia, with aberrant larval migration into the central nervous system causing eosinophilic meningoencephalomyelitis [60]. Parasitic infections, particularly those caused by *Cyathostomum* and *large Strongyles*, impair equine productivity by inducing anemia, weight loss, gastrointestinal disorders, and reduced nutrient absorption, ultimately compromising animal performance [39,40,61]. *Strongylus vulgaris*, known as the equine bloodworm, is one of the most dangerous gastrointestinal parasites, causing severe damage to the intestines, leading to colic, anemia, weight loss, and potentially life-threatening conditions like intestinal rupture [62]. *Parascaris equorum* infection in equines can cause respiratory signs during larval migration, poor growth, and malnutrition due to impaired nutrient absorption [63]; however, its main clinical manifestation is acute small intestinal obstruction (ascarid ileus), which may lead to severe colic and intestinal rupture if not managed promptly [64,65]. *Anoplocephala perfoliata* causes colic and induces pathological reactions at its attachment site, including hyperemia, mucosal thickening, and necrotic ulcers, which is why it is the only species linked to clinical diseases in horses [35,66]. Helminthic infections are widespread in equine populations, and their effects can be severe if left untreated, often leading to diminished health and productivity.

### 3.3. Ectoparasites and Vector-Borne Parasites

Ectoparasites and vector-borne parasites play a critical role in the transmission and pathogenesis of many equine parasitic diseases. These parasites include arthropod vectors (such as ticks, mites, flies, and mosquitoes) and the pathogens they transmit, which collectively contribute to dermatological issues, systemic diseases, and significant economic losses in equine operations [35,67,68,69]. Among these, the main insect vectors associated with *Setaria digitata* transmission are mosquitoes, particularly species of *Aedes, Culex*, and Anopheles, which act as intermediate hosts carrying infective larvae during blood feeding. Although ticks are not directly involved in the life cycle of *S. digitata*, other biting flies such as *tabanid* flies (*Tabanus* spp.) and stable flies (*Stomoxys calcitrans*) may serve as mechanical vectors for other equine parasitic or blood-borne infections [60,70,71,72,73]. Beyond their direct effects, such as skin damage and irritation, these parasites serve as vectors for bacteria, viruses, and protozoa, exacerbating their impact on equine health and productivity [27,35,67,68,69,74]. Ectoparasites significantly impair meat quality and carcass value. Tick and lice infestations cause skin lesions that downgrade hides, while botfly larvae (*Gasterophilus intestinalis*, *G. nasalis*) migrate through oral and gastrointestinal tissues, causing ulceration, reduced weight gain, and compromised carcass condition [75,76,77]. Ectoparasites and vector-borne diseases not only affect equine health directly but also complicate the control of parasitic infections by facilitating the transmission of other pathogens.

## 4. Effects of Parasitism on Equine Hematological Profile

Hematological parameters serve as important indicators of parasitic burden in equines, reflecting both direct parasite-induced damage and host-mediated immune responses [78,79]. Horses are susceptible to over 60 parasitic infections, such as nematodes, hemoparasites, intestinal parasites, and ectoparasites, which can significantly alter hematological parameters and impact overall health [80]. The most clinically relevant parasites, their associated diseases, and their hematological effects are summarized in Table 1.

Parasites such as *Babesia caballi*, *Theileria equi*, *Theileria haneyi*, and *Trypanosoma evansi* are major causative agents of hematological disturbances in equines. These infections commonly result in anemia characterized by reductions in red blood cell (RBC) count, hemoglobin (Hb), and hematocrit (HCT) due to hemolysis, as well as alterations in white blood cell (WBC) populations [25,47,81,82]. For instance, the intraerythrocytic parasitism caused by *Babesia caballi* in horses leads to hemolysis, which manifests clinically as severe hemolytic anemia, reduced RBC counts, and secondary hyperbilirubinemia (Camino et al., 2019), while *Theileria equi* and *Theileria haneyi* trigger erythrocyte damage combined with immune dysregulation, resulting in leukocytosis, mild anemia, and oxidative stress that further disrupt hepatorenal biomarkers [81,82]. Infections with *Trypanosoma evansi* (surra) similarly result in anemia with reduced RBCs, Hb, and HCT, alongside lymphocytopenia, monocytopenia, eosinopenia, and decreased antioxidant enzyme activities such as glutathione (GSH), superoxide dismutase (SOD), catalase (CAT), and total antioxidant capacity (TAC) [25,47]. These findings highlight that hemoparasitic infections primarily compromise erythrocyte integrity and antioxidant defense mechanisms, leading to varying degrees of anemia and systemic oxidative stress in affected equines.

Hematological alterations are also reported in equines infected with *Theileria annulata*, a hemoprotozoan parasite, and bacterial pathogens such as *Anaplasma phagocytophilum*, *Anaplasma marginale*, and *Neorickettsia risticii*, which are key contributors to the broader equine infectome [83,84,85,86]. *A. phagocytophilum* causes equine granulocytic anaplasmosis (EGA), an emerging tick-borne disease that induces fever, thrombocytopenia, leukopenia, and anemia due to granulocyte infection and immune-mediated hemolysis [84,87]. *A. marginale* infection is associated with erythrocyte destruction, anemia, and leukocytosis, whereas *Ehrlichia* spp. produce lymphocytosis and thrombocytopenia, collectively leading to substantial hematological and biochemical imbalance [88,89,90].

Donkeys exhibit anemia characterized by decreased hemoglobin and erythrocyte indices, alongside compensatory increases in hematocrit and monocyte counts, reflecting species-specific immune modulation. In contrast, horses, the infection induces leukocytosis and shifts in erythrocyte indices, consistent with hemolytic anemia [83,85,91]. Although *Theileria annulata* primarily infects bovines, recent molecular evidence has identified its occurrence in equines, where infection has been associated with elevated lymphocytes and MCH in horses, and increased lymphocytes and RBC counts but reduced hemoglobin and MCV in donkeys, indicating anemia and immune activation [83,84]. Similarly, *T. annulata* infection induces elevated lymphocytes and MCH in horses, with donkeys showing increased lymphocytes and RBC count but reduced hemoglobin and mean corpuscular volume (MCV), indicating anemia and immune activation [83,92].

Furthermore, equine besnoitiosis is caused by *Besnoitia* spp. presents with clinical signs such as skin lesions and hematological abnormalities, including anemia, leukocytosis, eosinophilia, and lymphocytosis, and hypoalbuminemia, findings consistent across donkeys and cattle infected with related *Besnoitia* species [93,94,95,96]. Collectively, these hematological alterations reflect both parasite-specific and host-specific responses, underscoring the diagnostic value of blood profile changes in identifying and managing equine parasitic infections.

Gastrointestinal parasites pose significant challenges to livestock health and productivity, leading to substantial economic losses through increased morbidity, treatment costs, and reduced performance [97,98]. Among these, gastrointestinal nematodes, particularly *Strongylus* species, are known to induce hematological alterations such as decreased hemoglobin (Hb), packed cell volume (PCV), and red blood cell (RBC) counts, reflecting mild anemia [99]. These changes arise from the parasites’ hematophagous nature and their interference with nutrient absorption and erythropoiesis [100,101]. In small ruminants, infections caused by *Haemonchus contortus*, *Strongyloides* spp., *Moniezia* spp., and liver flukes like *Fasciola hepatica* have been shown to significantly disrupt hematological and biochemical profiles, resulting in anemia, metabolic imbalance, and diminished productivity [102]. Additionally, *Strongyles*, *Eimeria*, and *Moniezia* infections in goats are associated with reduced Hb, PCV, and mean corpuscular hemoglobin (MCH), with the most severe effects observed in young animals and during the winter season [103]. Despite these well-documented impacts, there remains a critical need for species-specific, seasonally focused studies in equines to fully elucidate the pathophysiological mechanisms and develop targeted control strategies against gastrointestinal parasitism.

**Table 1:** Hematological effects induced by parasitic infections in equines.

Parasite	Parasitic Nature	Disease/Condition	Effects on Host Hematological Profile	References
*Trypanosoma evansi*	Protozoan hemoparasite	Surra (Trypanosomosis)	Reduced RBC, HCT, and Hb (anemia); lymphocytopenia, monocytopenia, eosinopenia; oxidative stress	[25,47]
*Theileria annulata* & *Anaplasma marginale*	*T. annulata*: Hemoprotozoan parasite *A. marginale*: Obligate intracellular Gram-negative bacterial pathogen	Theileriosis & Anaplasmosis (tick-borne)	Donkeys: anemia with decrease ↓ Hb and erythrocyte indices, increase ↑ hematocrit & monocytes; Horses: leukocytosis with altered RBC indices (hemolytic anemia); *A. marginale* specifically → Horses: ↓ RBCs/monocytes, ↑ WBCs/lymphocytes; Donkeys: ↑ monocytes/hematocrit, ↓ Hb/MCHC	[83,85,91,104]
*Theileria equi*/*Babesia caballi*/*Theileria haneyi*	Intraerythrocytic protozoan	Equine Piroplasmosis (EP)	Leukocytosis, mild anemia, hyperbilirubinemia; chronic carriers show oxidative stress and hepatorenal dysfunction	[81,82,105,106]
*Theileria annulata* & *Anaplasma marginale*	Hemoprotozoan parasite (*T. annulata*); Obligate intracellular Gram-negative bacterium (*A. marginale*)	Theileriosis & Anaplasmosis (tick-borne)	Donkeys: anemia with ↓ Hb and erythrocyte indices, ↑ hematocrit & monocytes; Horses: leukocytosis with altered RBC indices (hemolytic anemia); *A. marginale* specifically → Horses: ↓ RBCs/monocytes, ↑ WBCs/lymphocytes; Donkeys: ↑ monocytes/hematocrit, ↓ Hb/MCHC	[83,85,91,104]
*Theileria annulata*	Tropical Theileriosis	Protozoan (Apicomplexa)	Horses: increased lymphocytes, MCH; decreased RBCs, MCV Donkeys: increased lymphocytes, monocytes, RBCs; decreased hemoglobin, MCV, MCH (anemia)	[83,104]
*Besnoitia* spp.	Besnoitiosis	Protozoan	Mild anemia; leukocytosis; eosinophilia; lymphocytosis; hypoalbuminemia; increased alkaline phosphatase	[93]
*Anaplasma* *phagocytophilum*	Obligate intracellular Gram-negative bacterium	Equine Granulocytic Anaplasmosis (EGA)	Thrombocytopenia; leukopenia; anemia; mediated hemolysis	[83,84,85]
*Strongylus* spp.	nematodes;	Equine strongylosis (Strongylosis)	Anemia (reduced hemoglobin, PCV, RBCs); impaired erythropoiesis due to nutrient competition	[99,100,101]
*Strongyles*, *Eimeria*, *Moniezia*	Gastrointestinal parasites (nematodes and protozoa)	Parasitic enteritis in goats	Reduced hemoglobin, packed cell volume (PCV), and mean corpuscular hemoglobin (MCH)	[103]
*Haemonchus contortus*, *Strongyloides* spp., *Moniezia* spp., *Fasciola hepatica*	Gastrointestinal nematodes and trematodes	Parasitic infections in small ruminants	Anemia, metabolic imbalance, and disrupted hematological and biochemical profiles	[102]
*Fasciola hepatica* (Liver Fluke)	Zoonotic trematode	Equine (Fascioliasis)	Anemia: Decreased RBC count and hemoglobin levels. - Immunosuppression: Reduced leukocyte and lymphocyte counts.	[107]
*Strongyles*, *Eimeria*, *Moniezia*	Gastrointestinal parasites (nematodes and protozoa)	Parasitic enteritis in goats	Reduced hemoglobin, packed cell volume (PCV), and mean corpuscular hemoglobin (MCH)	[103]

↓ = decrease; ↑ = increase.

## 5. Consequences of Parasitic Infections on Reproductive Parameters

### 5.1. Effects of Parasitism on Equine Semen Quality

Parasitic infections are one of the primary health problems that hinder equine breeding and production, leading to substantial economic losses [108]. Trypanosome infections, particularly *Trypanosoma equiperdum* (dourine), directly impair semen quality in stallions by inducing orchitis, sperm abnormalities, and reduced motility. Chronic infections such as Surra (*Trypanosoma evansi*) and Nagana (*Trypanosoma. brucei*/*vivax*) further compromise fertility through systemic wasting and immunosuppression [109,110]. *Trypanosoma congolense* infection in jacks impairs semen quality by inducing anemia and oxidative stress, which reduces testicular oxygenation, while hormonal disruption is characterized by decreased testosterone and increased prolactin, disrupting spermatogenesis and impairing semen quality [111]. *Setaria equina*, though usually asymptomatic in body cavities, can localize in the scrotum and spermatic cord, causing orchitis, edema, and necrosis changes that indicate a potential to impair spermatogenesis and semen quality despite lacking direct evidence on semen parameters [112].

Additionally, while of the evidence on trypanosomiasis comes from ruminant studies, the detrimental impact of *T. vivax* on reproduction, manifesting as reduced fertility, abortions, and agalactia, highlights the broader implications of trypanosomiasis across host species [113,114]. In bulls, *T. vivax* reduces libido and increases semen volume without affecting sperm concentration or motility [115]. Recent studies have shown a strong association between trypanosomiasis (caused by *Trypanosoma evansi* in camels) and hormonal alterations, as well as testicular lesions [116,117]. Furthermore, larvae of the nematodes *Habronema* spp. and *Draschia megastoma* can cause ulcerative granulomas on the penile and preputial mucocutaneous junctions in stallions, potentially leading to penile prolapse and reproductive disorders [118,119]. However, there is still a need for more research to explore the direct impact of these infections on semen quality in equines, particularly concerning hormonal and testicular changes. Understanding the full scope of these parasitic effects is crucial to improving breeding management and reproductive success in equines.

### 5.2. Effects of Parasitism on Equine Pregnancy

Pregnancy success is a critical determinant of reproductive efficiency and economic viability in equine breeding programs [120,121]. Parasitic infections can compromise pregnancy through direct fetal invasion, placental damage, systemic illness in the mare or jenny, or indirectly via hematological and metabolic disturbances [44,122,123]. Molecular evidence has demonstrated the transplacental transmission potential of protozoan parasites such as *Neospora* spp. and *Sarcocystis neurona*, detected in placental tissues and amniotic fluid, suggesting their role in abortion and reproductive failure [124,125]. Microsporidial infections caused by *Encephalitozoon* spp., although infrequent, have been linked to placentitis and abortion in mares [122,126,127]. Collectively, these findings underscore the need for further epidemiological and experimental studies to elucidate the prevalence, pathogenic mechanisms, and reproductive impact of protozoan and microsporidial infections in equines, along with the development of effective diagnostic, preventive, and control strategies. The effects of these parasites on semen quality and pregnancy are summarized in Table 2.

Equine piroplasmosis, caused primarily by *Theileria equi* (formerly *Babesia equi*) and *Babesia caballi*, is a significant tick-borne disease affecting horses worldwide, including in Egypt [128]. *Theileria equi*, in particular, is a major cause of abortion in endemic areas, with transplacental transmission resulting in the birth of infected foals that may develop clinical disease [129,130]. This parasitic infection leads to systemic illness, anemia, and reduced reproductive capacity, significantly affecting fertility in mares and causing economic losses. Infected mares may experience abortion, and foals born to infected mares may develop neonatal piroplasmosis [44,129,131]. In endemic areas, *T. equi* is recognized as a major cause of abortion and economic loss in equines [106,129,132,133,134]. *Theileria equi* is the major cause of equine abortions in endemic areas [129]. Additionally, *Neospora hughesi* has been confirmed as a cause of equine abortion, with tachyzoites transmitted transplacentally, inducing severe lesions such as necrotizing pneumonia, myocarditis, hepatitis, and placentitis, leading to fetal death [135]. Despite these findings, the mechanisms of transplacental transmission and the factors influencing abortion risk in naturally infected mares remain poorly understood, creating a significant research gap that needs to be addressed for improving reproductive management in endemic regions.

Other parasites, including *Neospora caninum*, a major cause of abortion in cattle, has been reported to induce reproductive and neurological disorders in horses [33,34]. *Halicephalobus* spp. represent an emerging concern in equine reproduction, as first confirmed cases demonstrated their ability to cross the placenta, directly infect the fetus, and induce repeated abortions [136]. Furthermore, Free-living amoebae, such as *Acanthamoeba hatchetti*, have been associated with equine placentitis, potentially transmitted via mechanical transport by setae or localized vascular invasion [137]. Moreover, intestinal parasites in donkeys, including Strongyles, pinworms, trichoStrongyles, ascarids, flukes (Fasciolidae), and tapeworms (Anoplocephalidae), can cause digestive disturbances, weight loss, colic, and, at high burdens, reproductive problems [138,139]. These findings highlight the broad spectrum of parasitic infections that can compromise equine fertility and emphasize the need for integrated parasite control strategies.

**Table 2:** Equine parasites and their effects of parasitism on equine semen and fertility.

Parasite	Parasitic Nature	Disease	Effects of Parasitism on Equine Semen/Fertility	References
*Trypanosoma congolense*	Hemoparasite (blood protozoan	Trypanosomosis	↓ Testosterone, ↑ Prolactin, anemia, oxidative stress → impaired spermatogenesis	[111]
*Neospora hughesi*	Protozoan	Equine abortion	Transplacental infection causes fetal death; it can reduce reproductive efficiency and fertility in mares	[135]
*Trypanosoma equiperdum*	Extracellular hemoparasite	(Equine) Dourine	Orchitis Sperm abnormalities Reduced motility	[109,110]
*Trypanosoma vivax,*	Hemoparasite (extracellular)	Trypanosomiasis	Reduced fertility, abortions, agalactia, impaired reproductive activity in males and females	[113,114,115]
*Habronema* spp./ *Draschia megastoma*	Nematode larval stage	Penile/preputial granulomas	Pain, mating difficulty, penile prolapse infertility risk	[118,119]
*Toxoplasma gondii*	Obligate intracellular protozoan	Donkey/Toxoplasmosis, reproductive dysfunction	abortion,	[140,141]
*Neospora caninum*	Protozoan parasite	Horses/Neosporosis	Abortion, fetal death, reproductive loss	[142]
*Encephalitozoon* spp.	Microsporidian	Placentitis, Abortion	Causes placentitis and subsequent abortion	[122,126,127,143]
*Encephalitozoon cuniculi*	Microsporidian	Necrotising Placentitis,	Reported in a case leading to necrotising placentitis and abortion at the final stage of gestation	[126]
*Babesia caballi*	Protozoan parasite, tick-borne (*Rhipicephalus* spp., *Amblyomma* spp., *Dermacentor* spp.)	Equine Piroplasmosis	Transplacental infection, abortion, or birth of infected foals (may show disease signs)	[129,130]
*Theileria equi*		Equine Piroplasmosis	Major cause of equine abortions in endemic areas; transplacental	[129,130,138]
*Neospora hughesi*	Protozoan, an intracellular parasite	Neosporosis	Causes equine abortion via transplacental tachyzoite transmission; induces necrotizing pneumonia, myocarditis, hepatitis, and placentitis, resulting in fetal death	[135]
*Acanthamoeba hatchetti*	Free-living amoeba	Placentitis	Can compromise pregnancy	[137]

↓ = decrease; ↑ = increase.

## 6. Parasitic Consequences on Productivity (Milk & Meat)

Parasitic infections can substantially impair the productivity and hygienic quality of equine-derived products, directly impacting public health and marketability [144,145,146]. In dairy donkeys, higher cyathostomin burdens eggs per gram (EPG > 1000) impair performance by reducing milk lactose and quantity, while elevating milk urea and pH. This trend is consistent with parasitology surveys, which link high cyathostomin prevalence to compromised milk production at the farm level [23,147]. Gastrointestinal nematodes, particularly *Strongyles*, are highly prevalent in dairy donkeys and likely in mares, where they compromise host health, reduce milk yield, and alter milk composition through nutrient competition and chronic inflammation [148]. Endoparasites such as *Toxoplasma gondii* are of particular concern, as they can contaminate donkey milk and meat, posing a zoonotic risk upon consumption [149,150]. The trematode *Fasciola hepatica*, which infects the liver and bile ducts of equines, causes reduced milk yield and compositional changes, including lowered butterfat and protein content [151,152,153]. While in dairy cattle and other livestock there is evidence that ectoparasite burden (especially ticks and biting flies) can lead to reduced milk yield and altered milk composition [154,155,156]. However, findings remain inconsistent, with some studies reporting no significant impact on milk parameters [157], indicating a need for controlled, longitudinal studies to clarify the magnitude and variability of productivity losses caused by liver fluke infection in equines. The overall effects of these parasites on milk productivity are summarized in Table 3.

In northern Kazakhstan, horses infected with *Sarcocystis bertrami* and *Sarcocystis fayeri* exhibited muscle inflammation and dystrophic changes, which adversely affected meat quality and demonstrated the impact of parasitic infestation on the nutritional and commercial value of equine meat [24]. *Trichinella* spp. infections in horses, though often subclinical, can reduce meat quality and have a clear zoonotic significance [158]. Beyond equines, several parasites in livestock species provide valuable comparative insights into productivity losses. In ruminants, Trypanosoma vivax infection reduces herd productivity through weight loss, diminished milk and meat production, and elevated mortality rates, accounting for up to 33% of livestock deaths in Ethiopia and reducing milk yield by 45% [159,160]. Economic losses are further amplified by bovine besnoitiosis, caused by *Besnoitia* besnoiti, which results in mortality, prolonged recovery, and reduced milk output [161,162,163]. While in dairy cattle and other livestock, there is evidence that ectoparasite burden (especially ticks and biting flies) can lead to reduced milk yield and altered milk composition [154,155,156]. Importantly, parasitic infections weaken host immunity, increasing susceptibility to secondary bacterial or viral infections, thereby compounding productivity losses [164]. Despite these recognized impacts in ruminants, corresponding large-scale epidemiological data on productivity losses in equines remain scarce, representing a critical gap for targeted control strategies.

Parasitic infections also compromise meat quality in equines and other livestock through direct tissue damage, visible lesions, and biochemical alterations. In Yunnan Province, the high seroprevalence of *T. gondii* in horses and donkeys raises concerns for meat safety, as consumption of infected horse meat, particularly raw meat from imported sources has been linked to severe human toxoplasmosis [141,144,145]. Among equine meat pathogens, *Sarcocystis* spp., especially *S. bertrami* and *S. fayeri*, are the most detrimental, forming cysts in muscle tissue that degrade nutritional value, alter texture, and present a zoonotic hazard [24,165]. Similar productivity and quality losses occur in cattle due to *Echinococcus granulosus*, *Fasciola* spp., *Taenia saginata*, and *Sarcocystis* spp., which lead to organ condemnation, reduced marketable meat, and economic losses [166,167,168]. In sheep, *Sarcocystis* infection alters flavor metabolite profiles, increasing bitterness and sourness while reducing desirable aromas due to disruptions in lipid and amino acid metabolism [169,170]. Despite evidence of substantial deterioration in meat quality, systematic studies quantifying the economic cost of these changes in equines are lacking. There is an urgent need to develop effective parasite control strategies to prevent zoonotic transmission, protect public health, and ensure the sustainability of the equine industry.

**Table 3:** Equine parasites and their consequences on milk and meat quality.

Parasite	Parasitic Nature	Disease	Effects of Parasitism on Milk and Meat Quality	References
*Strongyles* (e.g., *Cyathostomum* spp., *Strongylus* spp.)	Gastrointestinal nematodes	Strongylosis causes anemia, weight loss, and poor body condition	Indirectly reduces milk yield and alters composition by impairing nutritional status and metabolic balance.	[148]
*Sarcocystis bertrami* & *Sarcocystis fayeri*	Protozoan parasites forming sarcocysts in muscle tissue	Sarcocystosis	Cause muscle inflammation and dystrophic changes, reducing meat quality and potentially affecting animal productivity	[24]
*Cyathostomins* spp.	Gastrointestinal nematodes	Larval Cyathostominosis	Causes severe intestinal damage, weight loss, and emaciation, leading to poor body condition and reduced meat quality.	[39,40]
*Strongylus* spp. (*Strongyles)*	Nematode intestinal roundworm)	Strongylosis	Causes unthriftiness, anemia, colic, diarrhea, poor weight gain, the poor weight gain and body condition imply reduced meat quality and lower overall productivity.	[61]
*Cyathostomins*	Gastrointestinal nematodes (small *Strongyles*)	Cyathostomiasis	High burdens of eggs per gram (EPG >1000) reduce milk lactose and quantity, increase milk urea and pH, leading to compromised milk production at the farm level	[23,147]
*Toxoplasma gondii*	Protozoan parasite (zoonotic)	Toxoplasmosis	Contamination of milk and meat; zoonotic risk; potential public health hazard	[149,150]
*Theileria equi*	Tick-borne protozoan	Equine piroplasmosis	Causes acute infection leading to abortion in mares; economic losses in equine breeding	[106,129,132,133]
*Fasciola hepatica*	Trematode	Fasciolosis	Reduced milk yield, lower butterfat and protein content; economic losses due to liver condemnation	[151,152,153,157]
*Trypanosoma vivax*	Protozoan	Trypanosomiasis	Reduced milk and meat production, weight loss, mortality; significant herd productivity losses	[159,160]
*Besnoitia* besnoiti	Protozoan	Bovine besnoitiosis	Reduced milk production, prolonged recovery, weight loss; economic losses	[161,162,163]
*Sarcocystis bertrami*, *S. fayeri*	Protozoan	Sarcocystosis	Muscle cysts cause reduced nutritional value, altered texture, and zoonotic hazard	[24,165]
*Fasciola* spp. (in cattle)	Trematode	Fasciolosis	Liver condemnation, reduced meat yield; economic losses	[167]
*Taenia saginata*	Cestode	Cysticercosis	Visible cysts in muscle; meat condemnation; economic loss	[168]
*Sarcocystis* spp. (in sheep)	Protozoan	Sarcocystosis	Changes in flavor profile: increased bitterness/sourness, reduced desirable aroma; biochemical changes in muscle	[169,170]

## 7. Zoonotic Relevance

Some parasites possess zoonotic potential, thereby threatening human health through direct contact with infected animals, contaminated environments, or the consumption of contaminated milk and meat products [171,172]. The consumption of equine products is common in Eastern European and Asia countries [173,174]. Among the pathogens, Sarcocystis spp. and Toxoplasma gondii have been reported in equine meat and milk in several countries [150,175,176,177,178,179,180,181,182,183,184,185,186,187,188]. Consumption of raw or undercooked equine meat containing tissue cysts of *Sarcocystis* spp. has been implicated in food poisoning incidents in Japan, with the parasite releasing sarcotoxins that can significantly affect both human and animal health [177]. Furthermore, a survey in the United States detected *Sarcocystis* antibodies in 6.9% of horses, indicating that their meat could be a potential source of zoonotic infection [189]. Equine Parasites and their zoonotic Potential are described in Table 4.

Similarly, *Toxoplasma gondii* has been identified as another zoonotic concern in equines. *T. gondii* antibodies were detected in 6.4% of donkeys in the US, suggesting that both donkey milk and meat could be sources of human infection [150]. Supporting this, Mancianti et al. (2014) [149] detected *T. gondii* DNA in the blood (13.6%) and milk (6.8%) of seropositive donkeys in Italy, demonstrating that tachyzoites can be excreted in milk. These findings indicate that consumption of raw donkey milk or meat may constitute a plausible route of zoonotic transmission to humans [149]. These findings suggest that the consumption of raw donkey milk or meat may be a potential route for zoonotic transmission to humans. The zoonotic risks posed by these parasites underscore the need for rigorous food safety measures, particularly in regions where raw equine products are consumed.

The public health significance of equine toxoplasmosis has been reported in multiple countries. In France, human cases were described by Elbez-Rubinstein et al. (2009) [190] and Pomares et al. (2011) [145] reported three clinical cases linked to atypical *T. gondii* strains, likely acquired through ingestion of raw horse meat imported from Canada and Brazil. In Canada and Brazil, viable *T. gondii* has been detected in slaughtered horses, highlighting these countries as potential sources of contaminated meat entering European markets. In the Middle East, Al-Khalidi and Dubey (1979) and Shaapan and Ghazy (2007) reported isolation of viable *T. gondii* from horses [191,192]. In Europe, Evers et al. (2013) confirmed the presence of *T. gondii* in slaughtered horses [193], and Paştiu et al. (2015) [194] documented both seroprevalence and viable parasite isolation in Romanian horses. These findings collectively underscore the importance of considering equine toxoplasmosis as a public health concern, particularly in regions where horse meat is consumed [194]. Nevertheless, further investigations are required to determine the parasite load in horse meat and to confirm its potential as a significant source of human infection.

**Table 4:** Equine Parasitic Zoonotic Potential.

Parasite	Host Animal	Disease	Zoonotic Effect	Reference
*Sarcocystis* spp.	Equines (Horses)	Sarcocystosis	Consumption of raw or undercooked horse meat can cause food poisoning in humans; it demonstrates zoonotic potential	[177]
*Toxoplasma gondii*	Donkeys	Toxoplasmosis	Detected in the blood and milk of donkeys; consumption of raw milk or meat may transmit infection to humans.	[149,150]
*Toxoplasma gondii*	Equine (Horses)	Toxoplasmosis	Detected in horses; consumption of raw or undercooked horse meat is linked to human infection cases	[145,190,191,192,193,194]
*Trichinella* spp.	Horse	*Trichinellosis*	Often subclinical in horses, but reduces meat quality due to larval encystment; causes human trichinellosis when raw or undercooked horse meat is consumed; several large outbreaks in Europe confirm strong zoonotic potential	[158]
*Sarcocystis* spp.	Horse	Sarcocystosis	Consumption of raw or undercooked horse meat can cause food poisoning in humans; it demonstrates zoonotic potential	[177]
*Sarcocystis* spp. & *Toxoplasma gondii*	Equine	Toxoplasmosis, Sarcocystosis	Infectious equid meat with the highest prevalence in donkeys; humans may acquire infection through the consumption of contaminated meat, highlighting zoonotic risk	[195]
*Fasciola hepatica*	Equines (horses, donkeys, mules)	Equine fasciolosis (liver fluke infection)	Recognized zoonosis (humans infected via metacercariae)	[107,196]

## 8. Transmission of Parasitic Infections in Equines

Parasitic infections in equines occur through diverse transmission routes, depending on the parasite’s biology. Hemoparasites like *Trypanosoma equiperdum* are transmitted venereally during sexual intercourse via infected seminal fluid or genital mucosa, whereas *Trypanosoma evansi* spreads mechanically through biting flies (stable flies, horse flies) and tsetse flies in Africa; occasional transmission via milk or coitus has also been documented [197,198,199]. Nematodes such as *Habronema* spp. and *Draschia megastoma* are acquired orally when equids ingest infective L3 larvae deposited near lips or wounds by fly vectors, leading to gastric, cutaneous, or rare pulmonary infections [200,201,202]. Protozoans like *Toxoplasma gondii* infect equids through ingestion of sporulated oocysts from contaminated feed, water, or soil; transplacental transmission has also been reported, posing additional reproductive risks [145,203,204].

**Figure 1:**
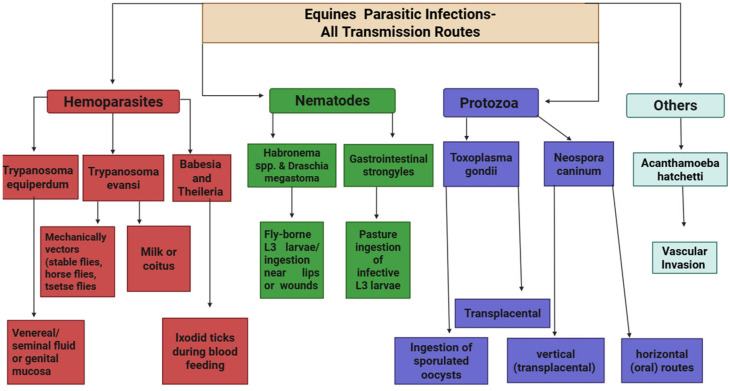
Schematic overview of major transmission pathways of equine parasites. Hemoparasites, nematodes, protozoa, and other parasites in equines are transmitted via venereal, mechanical, tick-borne, oral, transplacental, and vascular routes depending on species.

Blood-borne hemoparasites such as *Babesia caballi* and *Theileria equi* are transmitted by ixodid ticks during blood feeding, while *Neospora caninum* spreads via both vertical (transplacental) and horizontal (oral) routes depending on host species [27,205,206]. Free-living amoebae like *Acanthamoeba hatchetti* and caterpillar setae (e.g., Ochrogaster lunifer) can invade the reproductive system through vascular routes or migration from the gastrointestinal tract, leading to placentitis or abortion [207,208]. Gastrointestinal *Strongyles* follow direct life cycles, infecting equids via ingestion of infective L3 larvae from contaminated pasture [209]. Prevalence and control of strongyle nematode infections of horses in Sweden. Department of Biomedical Sciences and Veterinary Public Health, Swedish University of Agricultural Sciences [210]. The multifaceted transmission pathways (summarized in Figure 1) underscore the complexity of equine parasitic infections and highlight the critical need for detailed, species-specific epidemiological studies to quantify the relative importance of vertical, mechanical, and environmental transmission routes, particularly for emerging and zoonotic pathogens.

## 9. Diagnosis of Parasitic Infections in Equines

Accurate diagnosis of equine parasitic infections is essential for effective management. Hemoparasites like *Trypanosoma equiperdum* are primarily diagnosed through serological tests complement fixation test (CFT), enzyme-linked immunosorbent assay (ELISA), indirect fluorescent antibody test (IFAT), polymerase chain reaction (PCR) employed to differentiate closely related species (*T. equiperdum* vs. *T. evansi*) [211,212]. Similarly, equine piroplasmosis (*Babesia caballi* and *Theileria equi*) relies on microscopic examination of Giemsa-stained blood smears, serology (ELISA, IFAT, CFT), and highly sensitive molecular assays such as PCR for detecting low-level parasitemia and carrier states [213,214].

For gastrointestinal helminths, traditional coprological techniques such as fecal egg count (FEC), fecal flotation, and larval culture remain the cornerstone for diagnosis and monitoring of strongyle and ascarid infections in equines [215]. The McMaster and Mini-FLOTAC methods are widely used to quantify egg load and evaluate anthelmintic efficacy [216,217]. Serological assays, including an ELISA targeting Strongylus vulgaris antibodies, have improved early detection of migrating larvae and pre-patent infections [218]. Molecular diagnostic tools, particularly PCR-based assays, provide species-specific identification [219]. For gastrointestinal nematodes like *Habronema* and *Draschia*, traditional fecal egg counts and larval detection in tissue biopsies have limited sensitivity. PCR now provides a gold standard for detecting both gastric and cutaneous forms, enhancing early diagnosis and guiding targeted interventions [200,220]. Protozoan reproductive pathogens such as *Neospora caninum* and *Toxoplasma gondii* are diagnosed through combined serological (ELISA, IFAT) and molecular approaches (PCR targeting Nc5, ITS1, or *T. gondii* DNA), providing confirmation in mares and pre-colostral foals [221,222].

**Figure 2:**
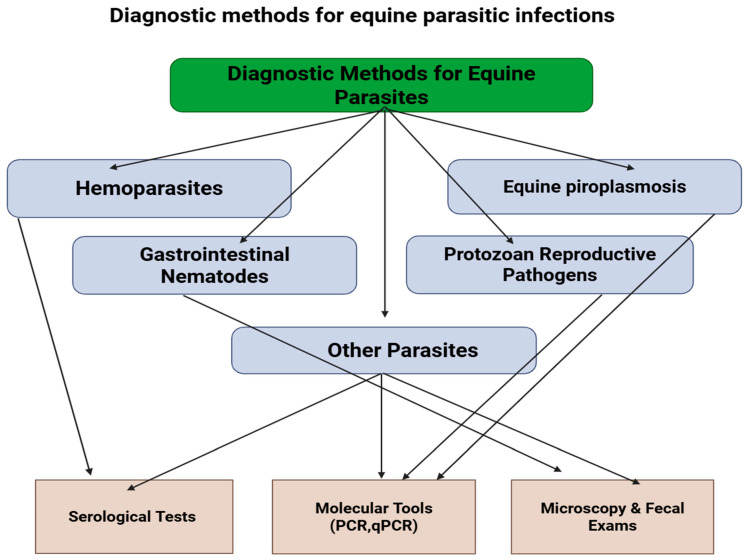
Flowchart demonstrating Overview of diagnostic approaches for major equine parasites, including hemoparasites, piroplasms, gastrointestinal nematodes, protozoan reproductive pathogens, and others. Commonly used tools include serological assays, molecular techniques (PCR, qPCR), and microscopy or fecal examinations, applied depending on parasite type and infection stage.

Other parasites, including *Encephalitozoon cuniculi* and *Acanthamoeba* spp., require specialized serological or molecular methods due to cryptic morphology and low parasitemia; conventional microscopy often fails to detect these pathogens reliably [137,223]. While molecular and serological methods have advanced diagnosis, there is a significant research gap in the development of rapid, field-applicable, and multiplex diagnostic tools capable of detecting multiple equine parasites simultaneously, especially for co-infections and early-stage reproductive pathogens (Figure 2).

## 10. Control and Treatment of Parasitic Infections in Equines

Control of equine parasitic infections requires integrated strategies combining chemotherapeutic, management, and vector control approaches. Hemoparasites like *Trypanosoma equiperdum* are treated with melarsomine (Cymelarsan^®^), though tissue-resident parasites may persist, necessitating strict OIE-recommended culling of infected animals in endemic areas [224,225]. Vector-borne nematodes (*Habronema* spp., *Draschia*) are managed with anthelmintics (ivermectin, moxidectin), fly control, and wound care to prevent recurrence of gastric or cutaneous lesions [226]. Table 5 summarizes the current evidence-based drug protocols employed for the control and treatment of key equine parasitic diseases.

For protozoan reproductive pathogens (*Toxoplasma gondii*, *Neospora caninum*, *Encephalitozoon cuniculi*), preventive measures such as feed hygiene, control of definitive hosts (cats, dogs), pasture management, and biosecurity are currently the most effective strategies, as no reliable chemotherapeutic treatments exist in equines [223,227]. Equine piroplasmosis (*Babesia caballi*, *Theileria equi*) is controlled through imidocarb dipropionate therapy, supportive care, and tick management; vector control remains essential to reduce transmission risk [228,229,230].

Helminth infections, particularly strongylosis, are managed through strategic anthelmintic administration combined with pasture hygiene, fecal removal, and rotational grazing; however, anthelmintic resistance is an emerging challenge requiring combination therapies and regular monitoring [215,231,232]. Similarly, equine besnoitiosis control focuses on preventing vector exposure and limiting contact with infected animals, as effective treatment options remain unavailable [233].

To minimize the development of anthelmintic resistance, deworming should follow targeted selective treatment (TST) based on fecal egg counts (FECs) rather than fixed calendar schedules [215,232]. Only horses showing high egg output (>200 EPG) should be treated, while low shedders remain untreated to maintain refugia [234]. Fecal egg count reduction tests (FECRT) every 6–12 months help monitor efficacy [235]. Accurate dosing according to body weight and rotating drug classes when efficacy declines are key for resistance prevention [236].

In addition to conventional chemotherapeutics, biological control approaches—particularly the use of the nematophagous fungus *Duddingtonia flagrans*, commercially formulated as BioWorma^®^—are emerging as sustainable alternatives in equine parasite management, effectively breaking the parasite life cycle by destroying gastrointestinal nematode larvae in feces and reducing pasture contamination when administered as a feed additive during the grazing season [237,238,239]. Similarly, *Bioverm^®^*, which also contains *Duddingtonia flagrans*, has been shown to reduce fecal egg count (EPG) and small strongyle larvae on pastures in horses. In a study, 16 mares were treated with *Bioverm^®^* at 1 g per 10 kg body weight daily for six months, resulting in improved weight gain and decreased parasitic burden [240]. Furthermore, *BioWorma^®^* (containing *Duddingtonia flagrans* NCIMB 30336) has demonstrated significant efficacy in reducing parasitic nematodes on pastures, improving the health of grazing animals, including horses. When used at a recommended daily application rate of 3 × 10^4^ chlamydospores per kg body weight, it effectively lowers parasitic burdens, offering a sustainable alternative to traditional anthelmintics [241]. In conclusion, both *BioWorma^®^* and *Bioverm^®^* offer effective, sustainable solutions for controlling parasitic nematodes in equines, improving their health, productivity.

Plant-based anthelmintics such as *Acacia nilotica*, *Rumex abyssinicus*, *Cucumis prophetarum*, *Allium sativum* (garlic), *Zingiber officinale* (ginger), *Chenopodium album*, and *Artemisia absinthium* have demonstrated significant antiparasitic potential against gastrointestinal nematodes [242]. Plant-based anthelmintics such as papaya latex, garlic, and neem extracts have demonstrated antiparasitic potential, though standardized dosing and efficacy studies are still limited in equines [243]. Currently, no standardized herbal anthelmintics or vaccines are available for equines due to limited efficacy validation, complex parasite biology, and insufficient research investment in the equine industry. Despite the development of various experimental vaccines, their application is hindered by challenges such as antigen identification, immune evasion by parasites, and the lack of large-scale, longitudinal studies [98]. Additionally, diagnostic and monitoring tools for equine parasites remain underdeveloped, further impeding the validation and widespread use of alternative treatments [244]. As highlighted in a systematic review, the effective control of gastrointestinal nematodes in horses is significantly delayed due to gaps in knowledge and research, compounded by the challenges posed by anthelmintic resistance in equines [80].

Anthelmintic resistance has become a global concern, particularly among cyathostomins and *Parascaris equorum*, with widespread reduced efficacy of benzimidazoles, pyrantel, and macrocyclic lactones [232,245]. Surveillance using fecal egg count reduction tests (FECRT) and molecular markers is now recommended to guide evidence-based deworming programs [38,246,247].

A recent study on equine parasite diagnostics and control notes that for protozoan parasites in horses, “no anthelmintic resistance (AR) mechanisms have been clearly elucidated” and “there remains a pressing need for immunological-based control and novel drug–target discovery” due to limited treatment options and absence of vaccines [244]. Future research should focus on developing novel therapeutics, integrating biological control, and employing predictive modeling of vector-borne parasite transmission to enhance preventive strategies.

Antibiotics remain indispensable for managing primary and secondary bacterial infections in horses; however, their use is often accompanied by adverse effects such as colitis, nephrotoxicity, neurotoxicity, arthropathy, and hypersensitivity reactions, and they can disrupt normal gut flora, predisposing animals to opportunistic infections [248]. Excessive or prolonged antibiotic administration has been shown to disturb metabolic balance and cecal microflora, reducing microbial diversity and beneficial metabolite production while promoting the proliferation of pathogenic bacteria such as Clostridium perfringens and *Salmonella* spp., which can lead to colic, diarrhea, and intestinal dysbiosis [249]. A large-scale survey of 14 UK equine practices (2012–2021) reported a median annual antibiotic usage of 54.25 mg/kg, with potentiated sulphonamides being the most frequently used class, while the use of critically important antimicrobials declined over time, reflecting progress in antimicrobial stewardship [250]. Overall, these findings highlight the need for careful and responsible antibiotic use in horses, along with regular monitoring, as published research on the long-term effects of antibiotics on equine health remains limited.

**Table 5:** Treatment of parasitic infections in equines.

Parasite/Disease	Drug & Dose	Route	Frequency/Protocol	References
*Trypanosoma* *evansi* (Surra) in horses & mules	Diminazene aceturate 3.5 mg/kg body weight	Intramuscular (IM)	On Day 0 and again on Day 41	[251]
*Trypanosoma* *equiperdum* (Dourine) in horses, acute & chronic infection	Melarsomine (Cymelarsan) 0.25 mg/kg and 0.5 mg/kg	Intramuscular (IM)	Single dose; also tested in repeated or comparative regimens	[224]
*Trypanosoma equiperdum* (Cymelarsan) in neurologic (CSF-positive) dourine	Melarsomine (Cymelarsan) 0.5 mg/kg daily for 7 days	route IM (used in study)	Repeated daily administration over 7 days	[252]
*Theileria equi* (Equine piroplasmosis)	Imidocarb dipropionate 4 Dose (mg/kg)	Intramuscular (IM), subcutaneous (SC)	Four injections 72 h apart	[253]
*Babesia caballi* (Equine piroplasmosis	Imidocarb dipropionate 2.2 mg/kg	IM/SC	Two injections 48 h apart	[254]
*Habronema*/*Draschia* spp. larvae	Ivermectin 0.2 mg/kg	Historically IM in study; commonly oral in practice	Single dose; lesions often need local care +/− anti-inflammatories	[255]
*Strongyles* (roundworms) and Tapeworms (*Anoplocephala perfoliata*)	Ivermectin (0.2 mg/kg) + Praziquantel (1.5 mg/kg)	Oral (paste formulation, Equimax^®^)	Single treatment suppressed strongyle FECs to zero for 10 weeks	[256]
*Cyathostomins* (small *Strongyles*, encysted larvae and adults)	Moxidectin 0.4 mg/kg	Oral (PO)	Single dose; reduced FEC by 99.9% at 14 days;	[257]
*Cyathostomins* (small *Strongyles*, encysted larvae and adults)	Fenbendazole 10 mg/kg	Oral (PO)	Once daily × 5 consecutive days; only 41.9% FEC reduction at 14 days;	[257]

## 11. Conclusions

Parasitic infections in equines present multifaceted challenges to both animal health and agricultural productivity, affecting various aspects, including hematological health, reproductive performance, and the quality of milk and meat. Protozoan parasites, particularly *Trypanosoma* spp., *Theileria equi*, and *Babesia caballi,* cause significant disruptions in semen quality, fertility, and pregnancy outcomes, often through direct transplacental transmission or by inducing systemic illness in mares and stallions. Helminthic parasites, including *Cyathostomins* and *Strongyles*, further exacerbate these issues by impairing nutrient absorption and contributing to reduced productivity. The zoonotic potential of equine parasites, especially *Sarcocystis* spp. and *Toxoplasma gondii*, highlights the need for stringent food safety measures to protect public health, particularly in regions where raw equine meat and milk are consumed.

Despite considerable advances in diagnostic and treatment methods, significant knowledge gaps remain regarding co-infections and their cumulative effects on animal health and productivity. Effective control requires integrated parasite management strategies combining targeted selective deworming based on fecal egg counts, rotation of anthelmintic classes, and pasture hygiene to minimize resistance development. However, no standardized herbal anthelmintics or vaccines are currently available due to limited efficacy validation, complex parasite biology, and insufficient research investment in the equine industry. As a limitation of the present review, a detailed phylogenetic illustration of protozoan parasites could not be included due to manuscript length constraints. Nevertheless, we recognize its importance and recommend that future research incorporate comprehensive phylogenetic analyses to better elucidate the evolutionary relationships among major equine protozoan parasites. Future research should also focus on developing novel therapeutics, herbal formulations, vaccines, and diagnostic tools, in conjunction with large-scale epidemiological investigations, to more accurately assess the prevalence and impact of parasitic infections in equines. Strengthening management, herbal, and research strategies will improve equine welfare and productivity while ensuring the sustainability of this vital sector.

## Data Availability

No new data were created or analyzed in this study.
